# Successful cannulation using a novel rotatable sphincterotome in a hepaticojejunal anastomotic stricture with a steep angle

**DOI:** 10.1055/a-2291-9720

**Published:** 2024-04-09

**Authors:** Yuki Tanisaka, Masafumi Mizuide, Akashi Fujita, Takahiro Shin, Kei Sugimoto, Ryuhei Jinushi, Shomei Ryozawa

**Affiliations:** 1 183786 Department of Gastroenterology, Saitama Medical University International Medical Center, Hidaka, Japan

Balloon enteroscopy-assisted endoscopic retrograde cholangiopancreatography (ERCP) is useful in patients with a hepaticojejunal anastomotic stricture 1 2 3 . However, biliary cannulation for such a stricture is challenging especially in cases with a steep angle, because the balloon enteroscope has no elevator function to angulate the cannulation catheter. We report a case of successful cannulation using a novel rotatable sphincterotome in a patient with a hepaticojejunal anastomotic stricture at a steep angle.

A 68-year-old man who underwent hepaticojejunostomy with Roux-en-Y owing to hilar bile duct cancer was referred to our facility because of recurrent cholangitis. Magnetic resonance imaging revealed a slight biliary dilation in the intrahepatic bile duct ( Fig. 1 ). Therefore, ERCP was performed using a short-type single-balloon enteroscope (SIF-H290; Olympus, Tokyo, Japan) with a working length of 152 cm and a working channel diameter of 3.2 mm 2 ( Video 1 ). After reaching the hepaticojejunal anastomosis, we observed the occurrence of a hepaticojejunal anastomotic stricture ( Fig. 2 ). Biliary cannulation using a conventional catheter was unsuccessful. The steep angle to the bile duct made biliary cannulation difficult. Therefore, we used a rotatable sphincterotome (Seeking Tome ZERO; MTW Endoskopie, Wesel, Germany) with a 1.8-mm tip diameter to achieve biliary cannulation ( Fig. 3 ). Guidewire seeking was attempted while angulating the sphincterotome. This allowed successful biliary cannulation ( Fig. 4 ). Cholangiography revealed the absence of stones. Subsequently, dilation of the stricture using a 4-mm dilation balloon catheter (REN; Kaneka, Tokyo, Japan) was performed, followed by plastic stent placement ( Fig. 5 ).

**Fig. 1:**
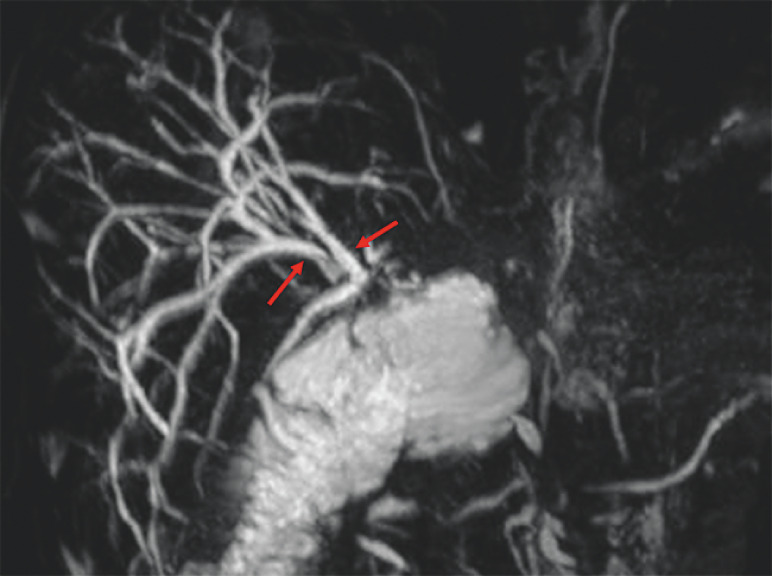
Magnetic resonance imaging revealing slight biliary dilation (red arrow) in the intrahepatic bile duct.

**Fig. 2:**
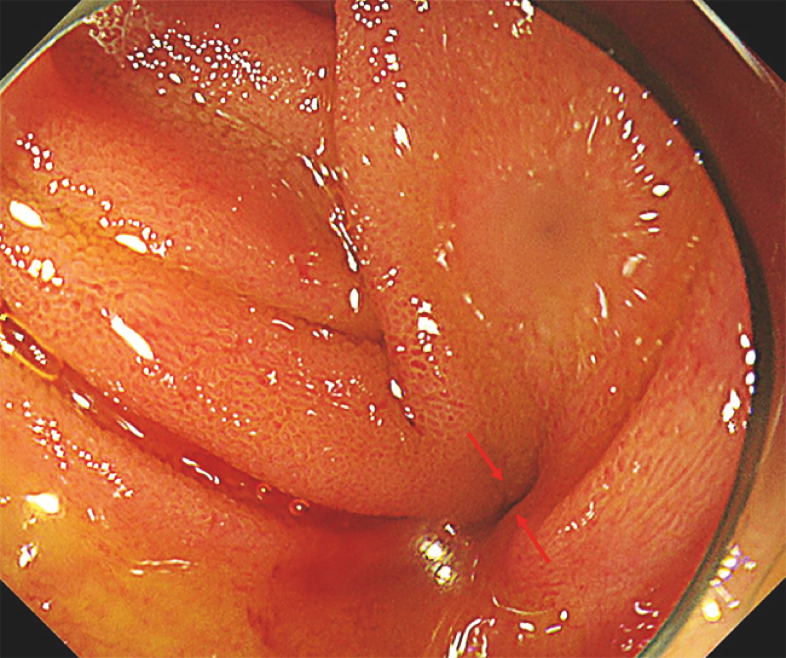
Endoscopic findings revealing the occurrence of a hepaticojejunal anastomotic stricture in the hepaticojejunal anastomosis (red arrow).

**Fig. 3:**
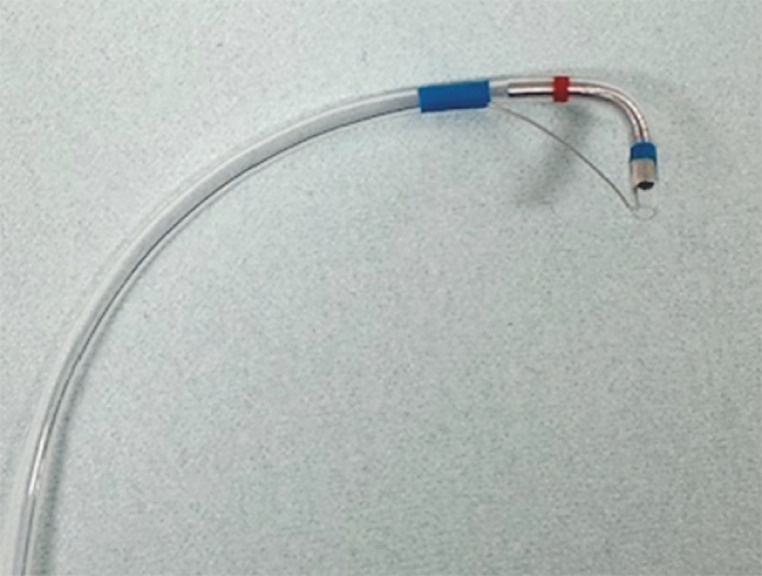
A novel rotatable sphincterotome with a tip diameter of 1.8 mm.

**Fig. 4:**
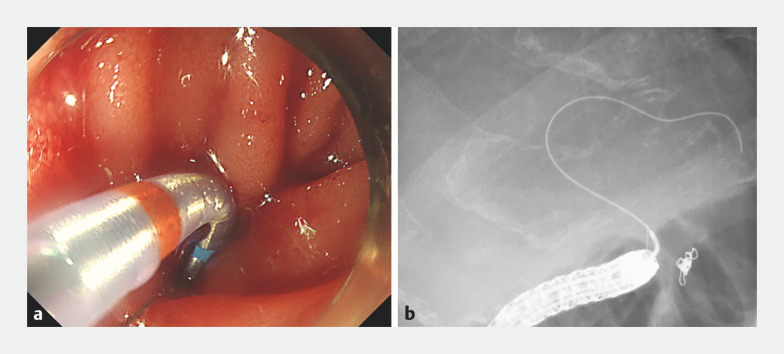
Endoscopic and fluoroscopic findings. Biliary cannulation is achieved by seeking the guidewire while angulating the sphincterotome.

**Fig. 5:**
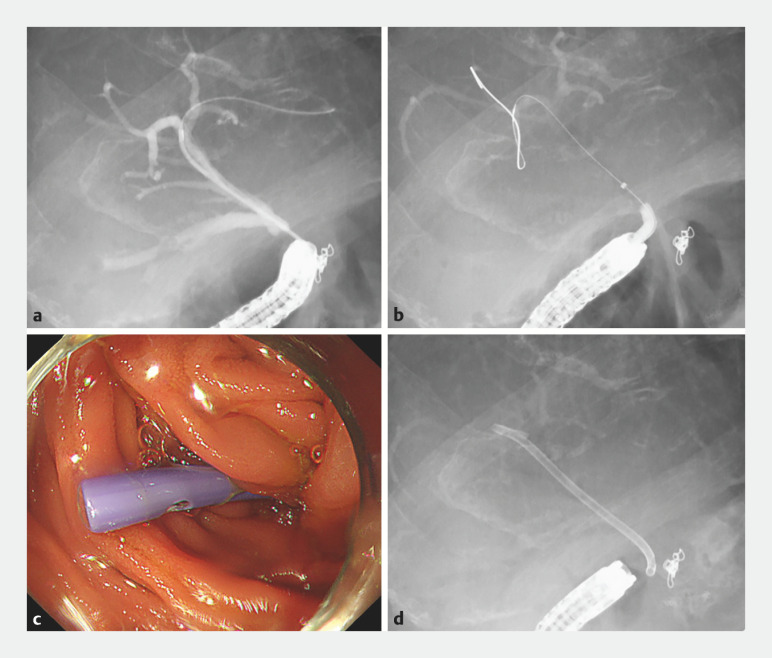
Endoscopic and fluoroscopic findings. **a** Cholangiography revealing the absence of stones. **b** Cholangiography revealing the dilation of the stricture using a 4-mm dilation balloon catheter. **c** , **d** Endoscopic findings and cholangiography showing the plastic stent placement.

Successful cannulation using a novel rotatable sphincterotome in a patient with a hepaticojejunal anastomotic stricture at a steep angle.Video 1

Compared to a previously reported rotatable sphincterotome 4 5 , this novel sphincterotome can be rotated even if there is hardly distance to the target site because it lacks a nose between the blade and the tip, which was helpful for our case. This novel rotatable sphincterotome can improve the success rate of biliary cannulation in difficult cases of hepaticojejunal anastomotic stricture.

Endoscopy_UCTN_Code_TTT_1AR_2AC

## Notes

Endoscopy E-Videos https://eref.thieme.de/e-videos
*E-Videos* is an open access online section of the journal *Endoscopy* , reporting on interesting cases and new techniques in gastroenterological endoscopy. All papers include a high-quality video and are published with a Creative Commons CC-BY license. Endoscopy E-Videos qualify for HINARI discounts and waivers and eligibility is automatically checked during the submission process. We grant 100% waivers to articles whose corresponding authors are based in Group A countries and 50% waivers to those who are based in Group B countries as classified by Research4Life (see: https://www.research4life.org/access/eligibility/ ). This section has its own submission website at https://mc.manuscriptcentral.com/e-videos .
